# External Built Residential Environment Characteristics that Affect Mental Health of Adults

**DOI:** 10.1007/s11524-013-9852-5

**Published:** 2014-01-25

**Authors:** Charles Ochodo, D. M. Ndetei, W. N. Moturi, J. O. Otieno

**Affiliations:** 1 Department of Environmental Science, Egerton University, P.O Box 536, Egerton, Njoro, Kenya; 2 Department of Psychiatry, University of Nairobi, Nairobi, Kenya; 3 Africa Mental Health Foundation (AMHF), Nairobi, Kenya; 4 Department of Geography, Egerton University, Egerton, Njoro, Kenya

**Keywords:** External built environment, Urban residential areas, Socioeconomic status, Psychosocial stress, Mental health disorders

## Abstract

External built residential environment characteristics include aspects of building design such as types of walls, doors and windows, green spaces, density of houses per unit area, and waste disposal facilities. Neighborhoods that are characterized by poor quality external built environment can contribute to psychosocial stress and increase the likelihood of mental health disorders. This study investigated the relationship between characteristics of external built residential environment and mental health disorders in selected residences of Nakuru Municipality, Kenya. External built residential environment characteristics were investigated for 544 residents living in different residential areas that were categorized by their socioeconomic status. Medically validated interview schedules were used to determine mental health of residents in the respective neighborhoods. The relationship between characteristics of the external built residential environment and mental health of residents was determined by multivariable logistic regression analyses and chi-square tests. The results show that walling materials used on buildings, density of dwelling units, state of street lighting, types of doors, states of roofs, and states of windows are some built external residential environment characteristics that affect mental health of adult males and females. Urban residential areas that are characterized by poor quality external built environment substantially expose the population to daily stressors and inconveniences that increase the likelihood of developing mental health disorders.

## Introduction

External built residential environment encompasses the built surrounding of dwelling units that provide the setting for human activities and range in scale from immediate to neighborhood and large-scale civic surroundings. On the other hand, internal built residential environment refers to the man-made components of the inside locations of dwelling units. The potential for built environments to promote health outcomes is linked to their effectiveness in facilitating stress coping and restoration. Good designs of the built environment can reduce anxiety, lower blood pressure, and reduce pain. Psychologically unsupportive surroundings are linked to negative effects such as higher occurrence of delirium, depression, and greater need for pain medication.^1^,^2^

Facility design should promote and maintain user independence, acceptability, convenience, and controllability. There should be a degree of confidentiality and privacy for occupants of a residential building particularly in toilets, bedrooms, and bathrooms.^3^ Access to external areas that promote a sense of normality through large windows, pleasant outdoor views, balconies, and courtyard areas are conducive to a healthy mental state.^3^,^4^

**FIGURE 1.**
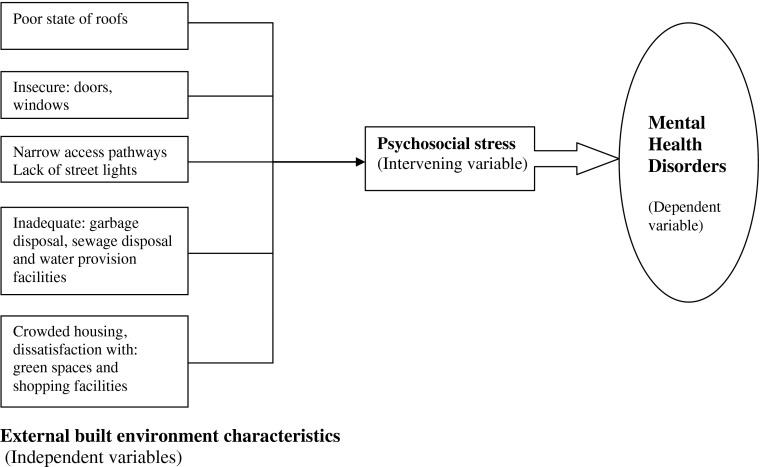
Conceptual model.

Residents who are continuously exposed to poor external built environment characteristics are likely to experience psychosocial stress that would in turn increase their likelihood of developing mental health disorders (Fig. 1). Mental health problems such as anxiety, depression, attention deficit hyperactivity disorder, substance abuse, and aggressive behavior relate to the built environment particularly to poor urban planning and inadequate housing.^5^,^6^ Dilapidated housing: leaking pipes, peeling paint, cracks, and holes in walls or ceilings are stressors that can adversely affect mental health.^7^,^8^

Inequities in construction and maintenance of low income housing result in insufficient housing, poor quality housing, overcrowding, higher levels of population density, and health problems.^6^,^9^ Housing designs that typically incorporate aspects of structural quality, maintenance, upkeep, amenities (such as private bathrooms, toilets, balconies), and prevention of physical hazards are particularly associated with good mental health. Humke et al. (1995) and Adam (2002) are of the view that insecurity often accompanies poor housing. The occupants of such housing are often low income earners who are concerned about housing tenure and constantly experience difficulties with repairs.^10^,^11^

Features of urban physical planning that enhance a sense of community include those which afford sufficient privacy, ensure residents have easier access to amenities, parks, recreation facilities, offer pedestrian friendly spaces, provide streetscapes so that houses have views of the surrounding neighborhood, encourage open verandas, and low fences and restrict motor traffic.^12^ The location of residential buildings in areas that limit availability and access to services can have adverse impacts on both physical and mental health. Seclusion has the potential of generating anxiety over accessibility to services.^13^,^14^

The immediate environment surrounding a building can influence health. Views of nature are particularly beneficial and are associated with less anxiety.^15^,^16^ Esthetic appeal including plants and flowers, landscaping, and artwork on walls are important for positive mental health outcomes. Residents appreciate good quality design as it makes them to be more relaxed.^17^ Relocation from low income neighborhoods to middle income areas is associated with enhanced mental health for both adults and children.^13^,^18^ The most frequently mentioned positive garden qualities are visual nature elements especially trees, greenery, flowers, and water. Respondents strongly associate these natural features with restorative influences on their moods.^19^ By contrast, a characteristic that usually lowers open spaces' effectiveness in reducing stress is a predominance of hard space such as concrete or starkly built content.^20^

Green spaces can have a positive impact on mental health through providing places for meetings and social interaction and places for exercise and relaxation. They provide pleasant visual experiences and improve air quality and, if occupied by trees, act as barriers for reduction of environmental noise and screen off particulate pollutants apart from absorbing carbon dioxide that is responsible for global warming.^21^ Encouraging green space development promotes a sense of community, reduces violence, and improves mental health.^21^

The quality of residential units is often related to the neighborhood in which they are situated. Poor quality housing is often located in neighborhoods with multiple indicators of urban decay such as dilapidated roads, vandalized utilities, graffiti, and garbage that can be harmful to health.^22^ Weich et al. (2002) found that housing areas with deck access had higher levels of depression.^23^ Deck access residences are multi-family units in which individual apartments open directly onto open walkways connected to a central staircase. These walkways are often public anonymous spaces with little evidence of residential social control or ownership. Such environments can impact on both mental and physical health through a reduction in physical activity, increased anxiety, and social disorder among residents.^23^–^26^ Wandersman et al. (1990) and Leventhal et al. (2000) argue that neighborhood quality has mental health impacts on children and their families independent of household socioeconomic status.^27^,^28^

Overcrowded housing conditions contribute to higher mortality rates, stress, infectious disease risk, and poor childhood development.^29^ Overcrowding is a major problem of the built environment especially in slums and squalid environments. It arises from poverty, overpopulation, and inefficient accommodation. It is a problem that has social and health effects.^30^ Chombant (1975) found that when each person had less than 8 to 10 m^2^ of space, then instances of physical illness and irregular behavior doubled compared to those in less crowded homes.^31^ Tripling of college students shows evidence of association between crowding and psychological distress. Similarly, residential density studies reveal evidence of psychological distress.^32^ Low class neighborhoods that were studied were often characterized by high population densities that exhibited tendencies of crowding both indoors and outdoors. Whereas residential density may not be related to psychological distress at initial occupancy; 6 months later, a positive correlation can be discerned.^33^,^34^

Low-cost housing in the developing countries like Kenya has peculiar features that may impact more on mental health than those of similar houses in the developed world. These features include poor structural designs that often consist of mud, iron sheets, or cardboard walls; lack of access roads or poorly kept pathways; absence of sewerage facilities and uncontrolled garbage dumping; lack of electricity connections and inadequate water supply. All these attributes impact negatively on mental health.

## Study Site

The location of the study was Nakuru Municipality which is one of the divisions within Nakuru County in Kenya. The municipality is located between longitudes 36 and 36°10′ E and latitude 0°10 and 0°20′ S at 1,859 m above the sea level. Nakuru is one of the fastest growing municipalities in Kenya; it is a modern and cosmopolitan town with several commercial centers. Its construction and transport industries are expanding rapidly.^35^ As a consequence of this growth, the population of the town has rapidly increased. According to the 2009 population census results, Nakuru town had a multiracial population of 224,743 people; 113,975 were males and 110,768 were females. The population density was 5,773 persons per square kilometer and there were 68,469 households.^36^ Most of the people live in high-density low income residential areas. Whereas many of the residents in the low income neighborhoods are tenants, a few of those in the middle income residential areas and most of the people in the high income areas have put up their own houses.^37^

## Method

This study was conducted through a cross-sectional social survey of residents in the low, middle, and high income neighborhoods of Nakuru Municipality in Kenya. Income levels of respective neighborhoods were determined by their type of housing that took into consideration types of walling and roofing materials, design of houses, and amenities as prescribed by the Kenya National Bureau of Statistics.^17^The total number of households in the four residential areas that were sampled (Milimani, Section 58, Shabab, and Rhonda) was 61,220.^36^

A total of 544 respondents from the respective residential areas representing the low, middle, and high income neighborhoods were selected through a multistage random sampling process. Among other factors, inclusion criteria depended on respondents' continuous residence in a given neighborhood for more than 1 year. This is because studies have shown that after living in a given neighborhood for more than 1 year, residents' exhibit behavior and health states that demonstrate either successful or unsuccessful adjustment to prevailing environmental characteristics.^38^,^39^ Nearly equal numbers of the low, middle, and high income households were interviewed because of stratification based on income levels. In all the three types of neighborhoods, different households that had at least one adult male or one adult female aged over 18 years or above were randomly selected. Only one member of each household who satisfied the preceding criteria was interviewed. In each category (males and females), a near equal number of respondents was interviewed.

The age range of respondents was 18–80 years, 274 were males and 270 were females. Out of the 274 males, 90, 96, and 88 belonged to the low, middle, and high income residential areas, respectively. As for the females, 95 were from the low income residential areas, 87 belonged to the middle income, and 88 were residents of the high income residential areas. Overall, 185 respondents were from the low income areas, 183 came from the middle income areas, and 176 belonged to the high income residential areas. Respondents were interviewed using structured questionnaires for environmental characteristics and validated lay mental health instruments.

## Mental Health Assessment

Mental health of respondents was assessed by the use of two instruments as follows: the Mini-International Neuropsychiatric Interview (M.I.N.I) plus and the Alcohol, Smoking, and Substance Involvement Screening Test. The M.I.N.I plus is a short structured diagnostic interview developed jointly by psychiatrists and clinicians for Diagnosis and Statistical Manual-IV and International Classification of Diseases-10 psychiatric disorders. In contrast to the usual clinical interviews, structured diagnostic interviews allow comparison across clinical centers or different environmental settings. In addition, they have the ability to reduce variability in diagnosis.^40^–^42^ The Alcohol, Smoking, and Substance Involvement Screening Test is an interview schedule that is designed to detect and manage substance use and related problems in primary and general medical care settings. It has undergone significant testing in three sequential phases (I, II, and III) to ascertain its psychometric properties.^43^

## Ethical Considerations

Ethical clearance for this study was obtained from the Kenyatta National Hospital Ethics Review Board. The nature and objectives of the study were explained to the respondents who voluntarily gave informed consent to be interviewed. Participants' names were coded to maintain confidentiality.

## Data Analysis

The external built environment characteristics across the three categories of residential areas, low, middle, and high income, were analyzed by the use of descriptive statistics, while chi-square tests (*P* ≤ 0.05) were used to determine the prevalence of specific mental health disorders of respondents. Mental health was assessed on the basis of one having lived in the neighborhood of residence continuously for the immediate preceding 12 months or longer.

Multivariable logistic regression models (*P* ≤ 0.05) were used to assess the relationship between the urban built external environment characteristics and the likelihood of developing specific mental health disorders. To avoid possible confounding, sociodemographic characteristics (age, sex, marital status, number of children and number of occupants in a household, daily food expenditure per person, level of education, type of employment, and type of residence) were controlled for in the models.

## Results

### Characteristics of External Built Environment

The external built environment characteristics studied were as follows: types of walls, states of roofs, heights of roofs above the ground, states of windows, types of windows, types of doors, access pathways for walking or vehicles, and street lighting. Others were garbage disposal facilities, sewage disposal facilities, pit latrines, external bathrooms, availability of green spaces, and water facilities (Table 1).

**TABLE 1:** Characteristics of the external built environment

External environment characteristics	(Residential areas)		
	Low income %	Middle income %	High income %
Housing units:			
With leaking roofs	74.1	13.7	12.2
With non-stone walls (mud/plastered mud)	100	0.0	0.0
Whose roof height Above the ground is less than 10 ft	98.5	1.5	0.0
Without recommended sizes of windows	100	0.0	0.0
Without secure windows	99.1	0.9	0.0
Without secure doors	73.5	20.3	6.1
Whose access pathways for walking are less than 2-m wide	66.3	31.8	2.0
Whose access pathways for vehicles are less than 6-m wide	78.2	20.9	0.9
With sufficient street lighting	3.0	49.2	47.7
Without garbage disposal facilities	97.7	2.2	0.0
Without sewage disposal facilities	98.6	1.4	0.0
With inadequate pit latrines	33.8	38.6^a^	27.6^a^
With inadequate bathrooms	99.6	0.4	0.0
Whose occupants walk beyond 500 m to access water	100	0.0	0.0
That are overcrowded	65.7	34.3	0.0
Residents who are dissatisfied with available green spaces	64.6	27.9	7.5
Residents who are dissatisfied with shopping facilities	47.4	7.4	45.3

^a^Houses had private flush toilets unlike the low income houses, 98.9 % of which lacked these toilets

### Height of Roofs

The low income houses had 98.5 % of roof heights that were less than 10 ft above the ground compared to the middle income ones that had 1.5 % roofs with such heights. The recommended height of roofs for residential houses should be at least 10 ft above the ground.^44^ Houses whose roof heights are less than 10 ft concentrate heat during the day and reduce indoor air circulation thus affecting ventilation. These attributes can engender chronic discomfort that may affect mental health of occupants.

### Windows and Doors

Whereas all housing units in the middle and high income residential areas had the recommended sizes of windows, none of those in the low income neighborhood had these windows (Table 1). In addition, 99.1 % of the windows in the low income houses were insecure owing to their wooden content. This sharply contrasted with the middle and high income ones that had only 0.9 % and no insecure windows, respectively.

Allied to windows were doors, 73.5 % of which were insecure in the low income houses. Only 20.3 and 6.1 %, respectively, of doors in the middle and high income houses were insecure. These categories of houses mostly had fences and hence did not experience the level of insecurity that characterized the low income ones.

The state, size, and types of windows and doors determine indoor temperatures and security of households. Wooden windows and doors were found to be less secure as they could be easily broken by solid objects. Families living in houses with such facilities were likely to experience anxiety and interrupted sleep. Lack of fencing, mud walls, and absence of bar grills on windows and doors increased vulnerability of occupants to external attacks. Households experienced discomfort particularly at night due to insecurity that they were exposed to owing to their types of doors and windows. Noise and intrusion from passers-by was not uncommon. Aggression among inhabitants was high in order to deter potential intruders. Suspicion of strangers was heightened and unwelcome remarks relayed in discourteous language were other manifestations of persistent discomfort.

Small wooden windows also reduced lighting and ventilation, thus contributing to high internal temperatures during daylight. Lack of windows or small windows is associated with higher levels of anxiety, depression, and delirium. According to Verdeberber and Leather et al., windows that allow enough light into the room are linked to favorable mental health outcomes.^45^,^46^

### Access Pathways

In the low income neighborhoods, 66.3 % of walking pathways were less than 2-m wide. As for access pathways for vehicles, 78.2 % of those in the low income residences were less than the recommended 6-m width, while in the high income residences, only 0.9 % of the vehicle access pathways were less than 6-m wide; this demonstrates the importance of motorized transport among high income residents.

Access pathways for walking and vehicles should be at least 2 and 6-m wide, respectively.^47^These pathways provide escape routes for residents in cases of emergency. Smaller pathways leading into dwelling environments are an indicator of overcrowding and can prompt feelings of claustrophobia. Where roads are less than 6-m wide, by-passing oncoming vehicles is not possible for drivers. In this case, they have to reverse, give way, or stop at junctions. This can be a security risk in some neighborhoods and may lead to mental distress. Narrow walking pathways also expose residents to inconveniences from bicycle and motorcycle taxis that are frequent in the low income neighborhoods and are common means of transport in the middle income residences.

### Street Lighting

The middle income neighborhood had the best street lighting rate with 49.2 % of the streets sufficiently lit at night. Overall, lack of street lighting or inadequate lighting was a notable problem in residential areas. Whereas most areas had not had any street lighting programs; some of the areas that were initially lit were plunged into darkness as a result of lack of maintenance of lighting systems or vandalism.

Pathways should be well lit at night. Dark or unlit roads are insecure in most neighborhoods. Such insecurity causes residents to worry when accessing the dark pathways. People are more likely to maximize the use of outdoor spaces if the neighborhood is perceived to be safe.^48^ Street lighting improvements show crime reduction effects and increase confidence of residents at night.^49^

### Garbage Disposal

In the low income residential areas where 97.7 % of households had no garbage disposal facilities, garbage was discarded anywhere outside the houses especially in pathways and drainage channels where wind would blow it to far off places. In the middle income residences, only 2.2 % of the households lacked garbage disposal facilities, those that had these facilities predominantly relied on paid up private garbage collectors. The latter were reported to be largely reliable. However, dogs and cats tore off the refuse paper bags placed outside residential gates thus scattering contents. Together with refuse from shopping premises that resulted from shoppers carelessly disposing wrappings such as polythene paper bags, the torn refuse bags contributed to unsightly garbage strewn in the vicinity of shopping premises and markets in the middle income residential areas.

The high income households all had garbage disposal facilities. Dug out pits into which garbage was dumped and periodically burnt were the predominant means of disposing or reducing garbage. The burning of garbage emitted smoke that polluted air, thus inconveniencing neighbors. Complaints arising from this practice were, however, few due to low population in the high income areas.

Even though a few high income households used private garbage collectors, they were not the preferred method mainly because of possible security breaches that they could occasion. The municipal authorities hardly collected garbage in all the three residential areas; this happened in spite of the residents regularly paying land rates that entitled them to services such as garbage collection.

Garbage or refuse collection facilities in residential areas should be adequate. Garbage should be removed from the dwelling units daily and disposed of in a sanitary manner. Disposal sites should be located on the leeward side and have a 100 m protection belt preferably comprising of trees.^47^ Garbage dump sites within or near residential units are an eyesore, emit offensive odors, and provide breeding grounds for organisms that cause or transmit diseases.

Quite often in the low income neighborhoods where dump sites were located near houses, wind blew light garbage such as paper bags to the door steps. Dogs or cats could also carry certain rotting edible garbage to residences. In a number of instances, slum residents relieved themselves in garbage dump sites particularly when they were located close to pit latrines.

### Sewage Disposal

Many of the low income housing units (98.6 %) had no effective sewage disposal facilities; they mostly relied on poorly constructed pit latrines. However, if they had enough water, adequate sewage disposal facilities linked to flush toilets would have served them better since the few pit latrines they had easily filled up due to large numbers of tenants. The middle income dwelling units were mainly connected to the municipal sewer line, while the high income residences mostly used septic tanks. Major problems with the municipal sewer line were recurrent blockages that led to sewage overflowing on roads and walk ways. This overflow sometimes found its way into housing compounds. In addition, the environment had a persistent offensive smell that affected residents. Sewage disposal and treatment plants are recommended for all settlements with a population of 3,000 people or more. In residences where an integrated sewage system is not available, septic tanks should be used.^47^

### Water Facilities

The low income families had no water facilities within their housing units; water taps were either located next to their houses or at central points to serve many households. Residents who had to walk beyond 500 m to access water were all within this income group (Table 1). In the informal settlements, watering facilities should be located within 500 m of dwelling units.^47^ With few far-placed water facilities, families spend long and stressful hours collecting water. Less water is drawn for household chores and this affects hygiene levels. Extended hours spent standing in the sun and dirty environments strain patience particularly where quantities of water decline and some residents foresee that they could miss it altogether. Usually, water brawls ensue at watering points that serve many households. Mothers who cannot afford maids worry about small children left back in the house on their own as they can suffer harm.

### Overcrowding

Overcrowding characterized 65.7 % of housing units in the low income neighborhoods. Overcrowding was also noted in 34.3 % of the middle income households; frequently, high-rise flats were crammed into a quarter of an acre of land leaving no room for vehicle parks or wash lines. The high income housing units were singly placed in their compounds and hence did not exhibit the features associated with overcrowding; however, residents complained of loneliness and less social interaction even during periods of bereavement. Ironically, they were visited more by their friends and relatives from the low and middle income neighborhoods. In many instances, neighbors in the high income residential areas including next door ones were not familiar with each other, neither did they express willingness to know each other.

Population density and the number of dwelling units in a given area determine overcrowding. For multifamily housing units, the recommended number of houses per hectare is as follows: low-density—50, medium-density—60, high-density—70, and extreme high-density—133.^47^ Informal settlements invariably exceed 400 dwelling units per hectare. 47 Overcrowding of dwelling units cause distress to residents as amenities such as latrines, bathrooms, and water are severely deficient. There is lack of space for movement or airing clothes. Littering of neighbors' frontages occurs, noise pollution, internal insecurity, and recurrent disagreements, e.g., due to activities of children are common. Quite often, neighborhood brawls resulted. This created fear among minors to the extent that some of them suffered from severe separation anxiety disorder. They felt extremely insecure when their parents or guardians were away or when they had to leave home for other places like school.

An association exists between poor mental health and lack of space within the home as well as lack of social space for interaction outside the home.^50^ Multi-occupation dwellings and flats, particularly high rise flats, are some of the housing risk factors associated with poor mental health.^51^

### Green Spaces

In the low income residential areas, 64.6 % of the population was dissatisfied with available green spaces compared to 27.9 % of those in the middle income and only 7.5 % of the high income ones. In the middle and high income neighborhoods, concrete compounds replaced green spaces while in the low income residential areas, open grounds that generated choking dust in the dry season or gave rise to muddy pathways dominated the small restricted compounds.

Green spaces provide esthetic appeal; grass and flower gardens denote tranquility. According to Ulrich (2002), viewing of plants and flowers ameliorates stress within 5 minutes or less.^52^ Viewing nature for longer periods helps to calm residents and can foster improvement in mental health. Green vegetation was found to promote positive attention influence on both females and males even though females manifested stronger influence.^52^

### Shopping Facilities

Shopping facilities were not a major source of distress to most of the residents in the middle income category where only 7.4 % of the households expressed dissatisfaction with the available shops. The low level of dissatisfaction with shopping facilities among the middle income residents was attributed to the availability of shops and supermarkets in the vicinity of their dwelling units. In addition, they had the necessary financial capability to purchase most of the goods in standard packaging sizes.

On the other hand, 47.4 % of residents in the low income neighborhood and 45.3 % of those in the high income residential areas expressed dissatisfaction with shopping facilities but for different reasons. The high income residents cited the far placed supermarkets and shops in town where they obtained most of their supplies as being inconvenient. While the low income residents who had shops and grocery kiosks very close to their door steps could not find certain items they required; the usual packaging of commodities like flour or sugar also appeared to be unaffordable to some of them. Shopkeepers therefore had to repackage items in smaller units.

### Prevalence of Mental Health Disorders among the Adult Residents of the Low, Middle, and High Income Neighborhoods

#### Depression

There was a significant difference in the levels of depression across the three income groups (Table 2). However, between the low and middle income neighborhoods, the level of depression was not significantly different (χ^2^ = 2.452, *P* = 0.117). Likewise, between the middle and high income residential areas, there was no significant difference in the prevalence of depression (χ^2^ = 2.244, *P* = 0.134). The low income neighborhoods had significantly higher levels of depression than the high income residential areas (χ^2^ = 9.164, *P* = 0.002). This implies that environmental characteristics that are associated with depression were significantly different in the two residential set ups. Of the females interviewed, 29.3 % expressed feelings consistent with depression compared to 19.7 % of the men. On average, 24.5 % prevalence of depression was recorded among the residents of the three neighborhoods.

**TABLE 2:** Prevalence of mental health disorders among adults in the low, middle, and high income residential areas

Mental health disorder	(Residential area)			Chi-square (χ^2^)	*P* ≤ 0.05
	Low income %	Middle income %	High income %		
Depression	31.4	24	17.6	9.240	0.01
Dysthymia	3.2	2.7	8.5	8.064	0.02
BMD	2.2	1.1	3.4	2.234	0.33
Panic disorder	8.1	1.6	9.7	10.891	0.004
PTSD	8.7	1.6	6.3	8.944	0.01
AA&D	0.5	6.0	2.8	9.163	0.01
GAD	7.0	1.6	14.2	20.431	0.00

*BMD* bipolar mood disorder, *PTSD* post-traumatic stress disorder, *AA&D* alcohol abuse and dependence, *GAD* generalized anxiety disorder

Type of walling material, access pathways for walking, and availability of shopping facilities were significantly associated with depression (Table 3). Respondents who lived in houses built of stones were 0.093 times less likely to experience depression in comparison to those who lived in non-stone wall houses. Residents who had access foot paths measuring more than 2-m wide were 0.592 times less likely to develop depression. Those who expressed dissatisfaction with shopping facilities were 2.307 times more likely to suffer from depression compared to those who were satisfied with these facilities. The significant confounders that were associated with depression were sex, type of residence (tenant or own house), and level of education.

**TABLE 3:** Relationship between the external built environment characteristics and mental health disorders: multivariable logistic regression analyses

Mental health disorders	Characteristics of external built environment	Exp (β)	95.0 % CI for Exp (β)		*P* ≤ 0.05
			Lower	Upper	
Depression	Type of walling material	0.093	0.009	0.967	0.047
	Access pathways for walking	0.592	0.354	0.990	0.046
	Shopping facilities	2.307	1.049	3.955	0.036
Bipolar mood disorder	Type of doors	0.113	0.016	0.772	0.026
Panic disorder	State of roof	0.294	0.106	0.8I7	0.019
	State of windows	8.344	1.975	35.248	0.004
	Functional bathrooms	0.089	0.009	0.918	0.042
	Dissatisfied with available green spaces	4.262	1.215	14.945	0.024
Post-traumatic stress disorder	State of roof	0.353	0.137	0.911	0.031
	Distance traveled to access water	4.760	1.791	12.368	0.002
Alcohol abuse and dependence	Type of doors	0.081	0.011	0.600	0.014
	Access pathways for walking	386.4256	5.578	26767.992	0.006
	Lack of street lighting	15.623	1.921	127.059	0.010
	Dissatisfaction with available green spaces	0.002	0.000	0.125	0.003
Generalized anxiety disorder	State of roof	0.322	0.122	0.850	0.022
	Distance traveled to access water	5.327	1.855	15.299	0.002

*All models were adjusted for age, sex, marital status, number of children, number of occupants in a house, level of education, employment, and type of residence

According to Ndetei et al., depression is a common mental health disorder that affects people of all races, socioeconomic, and cultural backgrounds.^53^ It is important not only because of lost productivity and the pain it causes to the affected individuals and their families but also due to its cost to human life through suicidal attempts or actual suicide. Depression affects more women than men. Major depression is manifested by a combination of symptoms that are severe enough to interfere with the ability to work, sleep, eat, and enjoy pleasurable activities. It can occur once, twice, or several times in a lifetime.^53^

#### Bipolar Mood Disorder

The overall incidence of this disorder in the three neighborhoods was 2.2 % similar to that in the low income class. However, the middle income residents had a lower level of 1.1 % unlike the high income ones who expressed the highest level of occurrence at 3.4 %. There was no significant variation in the prevalence of this disorder across the three categories of residential areas neither did any two of these residential areas express a significant difference in the level of bipolar mood disorder. Likewise, there was no significant variation in the disorder between males and females who manifested a rating of 1.8 and 2.6 %, respectively.

Bipolar mood disorder was significantly associated with one characteristic of the external environment. Residents whose house doors were made of steel were found to be 0.113 times less susceptible to bipolar mood disorder than those whose doors were wooden (Table 3). The significant sociodemographic factors that were associated with bipolar mood disorder were being employed or unemployed and type of residence.

Bipolar disorder also known as manic depressive disorder is a psychiatric diagnosis that describes a category of mood disorders defined by the presence of one or more episodes of abnormally elevated energy levels, cognition and mood with or without one or more depressive episodes. The elevated moods are clinically referred to as mania or if milder, hypomania. Persons affected by manic episodes commonly experience depression.^54^

#### Panic Disorder

Panic disorder was highest among the high and low income residents: 9.7 and 8.1 %, respectively, while males and females were equally affected. Overall, 6.4 % of the respondents experienced the disorder. Like the low and middle income residential areas which indicated a significant difference in the levels of panic disorder (χ^2^ = 8.275, *P* = 0.004), the middle and high income residential areas also had a significant variation in the prevalence of this disorder (χ^2^ = 10.969, *P* = 0.001). However, there was no significant difference in the levels of panic disorder between the low and high income residential areas (χ^2^ = 0.269, *P* = 0.604).

Living in a house with very small windows increased the odds of experiencing panic disorder by 8.344 times as opposed to living in one with standard size windows. Residents who had adequate bathrooms reduced their chances of developing panic disorder due to lack of these facilities by 0.089 times. Dissatisfaction with available green spaces increased the chances of developing panic disorder by 4.262 times in contrast to satisfaction with these spaces. Level of education, daily food expenditure per person, and type of residence were significant sociodemographic characteristics that were associated with panic disorder.

Panic disorder is characterized by recurring severe panic attacks; sometimes, it is accompanied by significant behavior change lasting for a month or longer and on-going worry about the implications or concern about experiencing subsequent attacks also called anticipatory attacks. Panic attacks are entirely unpredictable, hence individuals become stressed, anxious, or worried wondering when the next attack will occur.^55^ The three known types of panic disorder are as follows: unexpected, situationally bounded, and situationally predisposed.^37^

#### Post-Traumatic Stress Disorder

Across the three income groups, the level of post-traumatic stress disorder (PTSD) was 5.5 %. The low income residents had the highest incidence recorded at 8.7 %, while its incidence among the high income residents was 6.3 %. The prevalence of this disorder was not significantly different between the low and high income residential areas (χ^2^ = 0.750, *P* = 0.387). However, between the low and middle income residential areas, there was a significant variation in the levels of PTSD (χ^2^ = 9.231, *P* = 0.002), likewise, a significant variation was recorded between the middle and high income residential areas (χ^2^ = 5.089, *P* = 0.024). Males and females expressed similar patterns of PTSD: 5.8 and 5.2 %, respectively.

Living in houses that had non-leaking roofs reduced susceptibility to PTSD by 0.353 times in relation to living in houses with leaking roofs. Residents who traveled more than 500 m to access household water increased their chances of experiencing PTSD by 4.760 units compared to those who accessed water within a distance of 500 m.

PTSD is a severe anxiety disorder that develops after exposure to any event that results into psychological trauma. This event may involve the threat of death to oneself or someone else or to ones or someone else's physical, sexual, or psychological integrity overwhelming the individual's ability to cope.^40^,^56^,^57^

#### Alcohol and Drug Abuse

Across the three categories of income, alcohol abuse and dependence was lowest in the low income residential areas (0.5 %) and highest in the middle income residential areas at 6.0 %. Whereas there was a significant variation in the level of alcohol abuse and dependence between the low and middle income residential areas (χ^2^ = 8.727, *P* = 0.003), there was no significant variation in the prevalence of this disorder between the low and high income residential areas (χ^2^ = 2.920, *P* = 0.087). Similarly, no significant difference was recorded between the middle and high income residential areas (χ^2^ = 2.117, *P* = 0.146).

Living in a house with steel doors reduced susceptibility to alcohol abuse by 0.081 times in comparison to living in a house with wooden doors. Houses with steel doors are more secure than those with wooden doors. Residents living in houses with wooden doors experience anxiety and fear of attacks by robbers or thieves at night. In order to assuage this fear, some of them turn to alcohol. Indeed, some residents that took alcohol in high quantities acquired temporary courage and even slept with their doors unlatched, some even spent the night on their doorsteps.

Residents living in areas with access footpaths that exceeded 2-m width were 386.426 times more prone to alcohol abuse and dependence than those who lived in areas with smaller access footpaths. Likewise, residents who lived in neighborhoods with sufficient street lighting were 15.623 times more likely to abuse alcohol than those who lived in areas with insufficient street lighting. Residents who were dissatisfied with green spaces in their compounds were 0.002 times less likely to abuse alcohol than those who were satisfied with these spaces. The significant confounders were number of children in a household, number of occupants of a household, age, and daily food expenditure per person.

Alcohol dependence is a condition that is characterized by harmful consequences of repeated alcohol use, a pattern of compulsive alcohol use, and physiological dependence on alcohol, i.e., tolerance or symptoms of alcohol withdrawal. This disorder is only diagnosed when these patterns of behavior become persistent, very disabling, or distressing.^57^,^58^

#### Generalized Anxiety Disorder

The level of generalized anxiety disorder (GAD) across the income groups was established to be 7.5 %. The level of this disorder was significantly different between every two of the three categories of incomes that were studied. The chi-square and *P* values for the respective pairs of residential areas being low and middle, 6.421, 0.011; low and high, 4.933, 0.026; and middle and high, 19.697, 0.000. Both males and females had a near equal GAD prevalence of 7.7 and 7.4 %, respectively. This finding is consistent with Cameron's assertion that in the developing nations; the prevalence of GAD is about equal in both sexes. Cameron also established that in the developed countries, women are two to three times more likely to suffer from GAD than men.^43^

The state of roof and distance traveled to access water were significantly associated with GAD. Residents who lived in houses with non-leaking roofs were 0.322 times less likely to experience GAD than those who lived in houses with leaking roofs. Those who walked more than 500 m to access water for household use were 5.327 times more likely to experience GAD compared to those who accessed water within a distance of less than 500 m. Number of children in a household, age, type of residence, marital status, and level of education were significant sociodemographic factors.

GAD exceeds the usual anxiety people experience every day. It is a chronic and exaggerated worry that is also accompanied by tension which is not provoked by any particular event. Affected individuals always anticipate disaster; often, they worry excessively about almost anything including health, money, family, or work, though most of the time it is difficult to justify the worry. The simple thought of getting through the day provokes anxiety. Affected people are aware that their anxiety is more intense than the situation warrants.^53^,^59^

## Discussion

This study assessed a wide range of characteristics of the external built residential environment found in poor developing countries against a number of specific mental health disorders that were identified among respondents in three distinct types of residential areas. The attributes that were significantly associated with mental health disorders were the type of walling material used on buildings, access pathways for walking whose sizes were related to residential housing densities, availability of shopping facilities, types of doors, states of roofs, and states of windows. Others were availability of functional bathrooms, dissatisfaction with available green spaces, lack of street lighting in the neighborhood, and distance traveled to access water.

Types of walling material determine the level of draught in a house and security of the occupants. Mud or iron sheet wall houses allow excess wind flow internally; this predisposes residents to flu and pneumonia. Such walls can be easily breached by attackers. The combination of too much cold and fear of attack contribute to troubled incomplete sleep. Repeated inadequate sleep can lead to disorientation that may develop into depression.

Narrow access footpaths characterize crowded residences and reduce the possibility of escape in case of misfortunes such as estate fires or floods. Residents develop feelings of being trapped-in that may lead to claustrophobia. Constant preoccupation with personal safety is a stress factor that creates anxiety and affects mental health.

In residential areas, shops mainly supply foodstuffs but may also provide items like medicines and objects of use in households, e.g., brooms, tooth brushes, and toothpaste. In Nakuru Municipality, like in the rest of Kenya, many of the residents tend to purchase food items almost on a daily basis. For instance, milk for breakfast may be purchased every morning while vegetables for supper may be acquired every evening. When shopping or market facilities are not conveniently placed, distress is likely to set in as residents search for these items.

Prevalence of bipolar mood disorder is dependent on environmental characteristics that vary in different categories of income groups and genetic factors that are randomly distributed in populations.^60^ Individual psychosocial attributes including harsh environmental conditions can interact with genetic dispositions to influence the development and course of this disorder.^61^ Wooden house doors are not secure hence residents living in houses with such doors experience endless fear of attack by thieves especially at night. The absence of secure fencing around the residences adds to the worry for personal safety. This insecurity constitutes a harsh environment that can interact with the residents' genetic factors to influence the development of bipolar mood disorder. Living in a house with small windows was significantly associated with panic disorder. Small windows limit alternative routes for escape in case of fire outbreaks or other misfortunes, they also cause stuffiness and induced darkness in the house as they do not allow effective ventilation and sufficient daylight indoors.

Bathrooms are necessary for personal hygiene particularly at the beginning and end of the day. Where they are inadequate, residents become restless as they await their turn to use them. Sometimes, they are critically inadequate and so residents have to bathe in the open or in their houses. Both of these situations create panic as personal privacy or safety is not guaranteed especially in crowded residential areas. Bathing indoors means that visitors are unwelcome for the time being; besides, other housemates must stay outdoors and if they happen to barge in, embarrassment can occur. This creates anxiety that can lead to panic disorder.

Leaking roofs cause dampness that can lead to pneumonia among the affected residents. Reports from the Rift Valley Provincial General Hospital in Kenya indicate that pneumonia is one of the leading killer diseases in the Central Rift Valley region particularly in the cold and wet season.^62^ Deaths of loved ones traumatize residents leading to post-traumatic stress disorder as the cold weather approaches. Leaking roofs lead to damage of property, interfere with sleep, and affect the health of residents. According to Gingles et al., damp houses are associated with fatigue, headache, chronic anxiety, and depression.^59^

In the low income residential areas, water taps are few and far between. Frequently, water brawls occur and can lead to fatalities. Conflicts that were at times fatal were witnessed in the low income residential areas in the aftermath of the 2007 General Elections in Kenya. According to residents, some of their kin were attacked and killed as they went out in search of water. The memories of these events were still vivid in their minds at the time of conducting this research and increased their likelihood of developing post-traumatic stress disorder.

Quite often, water was not enough and families were forced to use little of it while foregoing some activities that required its use. Anxiety arose from the possibility of missing it altogether even after spending long hours in winding queues. Children left in the houses unattended also contributed to anxiety as they could burn in the charcoal braziers that were left with blazing fires cooking the midday meals, they could also hurt themselves using objects in the house like sharp knives or poisonous chemicals stored within their reach.

Areas with standard footpaths and sufficient street lighting are likely to be inhabited by richer folks who possess extra disposable income that they can use to purchase alcohol. Conversely, most residents who were dissatisfied with available green spaces were found in the low income residential areas. Even though some of them did accept that they took alcohol, they could not afford to consume it in high quantities on a regular basis like their middle or high class counterparts. Their capacity to abuse alcohol was curtailed by default of lacking the financial means to access it.

Since it is the low income residential areas that were mainly affected by lack of physical planning and inadequate amenities, residents who lived there had a higher likelihood of suffering from various mental health disorders identified in this study. Even though many residents of the low income areas consumed alcohol, the possibility of alcohol abuse and dependence was found to be higher in the middle and high income neighborhoods owing to their enhanced purchasing power. Judicious taxation of this beverage can therefore check its consumption.

Lack of shopping facilities in the high income residential areas was significantly associated with occurrence of depression in spite of the residents' ability to buy food items in large quantities and refrigerate. This association can be explained by the eating habits of Kenyans who prefer fresh food items to refrigerated ones. As a result, living in high income residential areas places restrictions on their buying habits.

Galea et al. provide an explanation that may account for the association between characteristics of the urban built residential environment and mental health disorders.^38^ Their psychosocial stress explanation suggests that living in neighborhoods characterized by poor quality built environment is associated with psychosocial stress that in turn may contribute to mental health disorders. 63,64

Urban residential areas characterized by poor quality built environment substantially expose inhabitants to daily stressors and inconveniences that can lead to greater social strain and a higher likelihood of developing mental health disorders. It is also possible that densely populated urban residential areas amplify social deficiencies and stressors.^65^,^66^ Thus, high populations in areas characterized by deteriorating built environment characteristics can exacerbate both social and poor mental health consequences.

### Limitations of the Study

In this study, it is possible that certain multiple risk factors clustered at different levels thus becoming difficult to investigate. Factors occurring at the individual, household, and neighborhood levels could all have an impact on urban residents' mental health. Residents could disproportionately experience individual risk factors associated with mental illness such as unemployment that is associated with poverty. The study sample comprised of low, middle, and high income groups and the unemployed; this reduced bias arising from poverty. Income was defined by attributes of the external built environment. Therefore, the rich people who chose to live in neighborhoods with poor external built environment characteristics were exposed to the prevailing adverse environmental characteristics and could consequently experience poor mental health. Other confounding factors that could influence mental health were age, sex, period in residence, size of household, and house tenure (tenancy, leasehold, or freehold), all of which were individually analyzed in order to establish their relationship with mental health states of residents.

## Conclusion

This study demonstrates distinct associations between explicit characteristics of the urban external built residential environment and the likelihood of specific mental health disorders in adult males and females. It proves that low income residential areas have a higher concentration of poor external built environment characteristics and therefore expose residents to increased possibilities of developing mental health disorders. It is therefore necessary to improve qualities of the external built residential environment as part of key interventions to safeguard public mental health. Urban authorities can use available resources to upgrade the built residential environment. Such upgrading does not necessarily have to involve construction of new houses similar to those in the middle or high income residential areas. For a start, modification of available living structures to protect residents from the elements, decongesting residential estates by adhering to prescribed housing densities, provision of street lighting, sanitary, and garbage management facilities alongside maintenance of access pathways would suffice. These interventions call for close partnership between urban physical planners and public health professionals.

Since dense populations in low income neighborhoods aggravate social strain and consequent mental health disorders, easing of population pressure in these areas by stemming rural–urban migration and improving overall neighborhood economic outlook should be prioritized. Developing rural infrastructure alongside provision of basic amenities like electricity and water can encourage establishment of agro-based industries that would reduce rural–urban migration.
